# *LsMYB15* Regulates Bolting in Leaf Lettuce (*Lactuca sativa* L.) Under High-Temperature Stress

**DOI:** 10.3389/fpls.2022.921021

**Published:** 2022-06-28

**Authors:** Li Chen, Mengnan Xu, Chaojie Liu, Jinghong Hao, Shuangxi Fan, Yingyan Han

**Affiliations:** Beijing Key Laboratory of New Technology in Agricultural Application, National Demonstration Center for Experimental Plant Production Education, Beijing University of Agriculture, Beijing, China

**Keywords:** melatonin, leaf lettuce, high temperature, bolting, ABA-related gene

## Abstract

High temperature is one of the primary environmental stress factors affecting the bolting of leaf lettuce. To determine the potential role of melatonin in regulating high-temperature induced bolting in leaf lettuce (*Lactuca sativa* L.), we conducted melatonin treatment of the bolting-sensitive cultivar “S39.” The results showed that 100 μmol L^−1^ melatonin treatment significantly promoted growth, and melatonin treatment delayed high-temperature-induced bolting in lettuce. RNA-seq analysis revealed that the differentially expressed genes (DEGs) involved in “plant hormone signal transduction” and “phenylpropanoid biosynthesis” were significantly enriched during high-temperature and melatonin treatment. Quantitative reverse transcription polymerase chain reaction (qRT-PCR) analysis suggested that the expression patterns of abscisic acid (ABA)-related genes positively correlated with stem length during leaf lettuce development. Furthermore, weighted gene co-expression network analysis (WGCNA) demonstrated that MYB15 may play an important role in melatonin-induced resistance to high temperatures. Silencing the *LsMYB15* gene in leaf lettuce resulted in early bolting, and exogenous melatonin delayed early bolting in leaf lettuce at high temperatures. Our study provides valuable data for future studies of leaf lettuce quality.

## Introduction

Leaf lettuce (*Lactuca sativa* L.) is an annual vegetable crop of the Asteraceae family. It produces a range of organic compounds, such as proteins, fiber, phenols, and others, and has both edible and medicinal value (Chadwick et al., 2016). As the cultivation of leaf lettuce expands year on year and development continues, it has become one of the most-consumed leafy vegetables in the world (Viacava et al., 2017). Leaf lettuce is primarily cultivated in polytunnels and similar facilities and is prone to bolting (producing a flowering stem prematurely) at high temperatures during cultivation. Bolting is accelerated under high-temperature conditions, which causes leaves to become bitter-tasting and limits crop marketability (Han et al., 2021a,b). Therefore, delayed bolting and flowering are preferred in lettuce for maximizing harvestable yield while maintaining culinary quality.

Leaf lettuce is a cruciferous vegetable and self-pollinating plant whose flowering is accelerated under longer day lengths and higher ambient temperatures (Uwimana et al., 2012). Premature flowering limits vegetative growth, and thus the accumulation of sufficient resources for sustained growth. Plants undergo profound physiological changes when transitioning from vegetative to reproductive growth. Due to its importance in both basic and translational biology, flowering time has been extensively studied in model plant species, particularly in *Arabidopsis thaliana* (thale cress; Han et al., 2021a,b). Research has shown that, in *Arabidopsis*, at least six genetic pathways regulate the transition to flowering, including the photoperiod, vernalization, autonomous, ambient temperature, age-dependent, and gibberellin (GA) pathways, which coordinate with one another to regulate flowering (Izawa, 2020). The signals from flowering pathways, such as *FLOWERING LOCUS T* (*FT*), *SUPPRESSOR OF OVEREXPRESSION OF CO1* (*SOC1*), and *LEAFY* (*LFY*), are integrated into the genetic network of flowering (Jutarou et al., 2021). Among these integrons, the florigen gene *FT* is the central node of floral transition, whose transcriptional expression is positively regulated by zinc finger transcription factor *CONSTANS* (*CO*), while negatively regulated by *FLOWERING LOCUS C* (*FLC*; Zong et al., 2021). It has been widely reported that different environmental factors affect plant flowering by regulating the expression of floral integrons and stimulating changes in plant hormone levels (Zou et al., 2020). Increasing evidence suggests that flowering time is associated with plant hormones (Ying et al., 2014). In *Arabidopsis*, it has been found that the target of the repressive function of abscisic acid (ABA) is the flowering-promoting gene *SOC1*. One report has it that the CORONATINE INSENSITIVE1 (COI1)-dependent signaling pathway delays the flowering time by inhibiting the expression of the florigen gene FLOWERING *LOCUS T* (*FT*). This proved that the APETALA2 transcription factor (TFs) TARGET OF EAT1 (TOE1) and TOE2 interact with JAZ (Jasmonate-Zim domain) protein and repress the transcription of FT (Zhai et al., 2015). Therefore, understanding the crosstalk between hormones and flowering-related genes is of great importance for elucidating the regulatory mechanisms of bolting and flowering in plants.

In vegetable cultivation and production, leafy vegetables that can bolt include, primarily, Chinese cabbage, beets, spinach, celery, onion, and lettuce. The “low-temperature vernalization” types of cruciferous vegetables have been extensively studied. In *Arabidopsis*, *FRIGIDA* (*FRI*) and *FLOWERING LOCUS C* (*FLC*) are important genes regulating the vernalization pathway, and mechanistically interact to control flowering time (Schon et al., 2021). *FLC* acts as a strong repressor of floral transition; before vernalization, expression of *FLC* is increased, and plants cannot bloom. When plant vernalization is completed, expression of *FLC* decreases, and the plants bloom. In Chinese cabbage (*Brassica rapa* ssp. *chinensis*), *BcFLC2* plays a key role in flowering regulation as a negative regulator by controlling the expression of *BcSOC1*, *BcSPL15*, *BcMAF2*, *BcTEM1*, and other genes closely related to flowering (Huang et al., 2018). The vernalization response in biennial beets (*Beta vulgaris*) is mediated by the *FT* homolog *BvFT1*, which, in contrast to the promotive action of *FT* in *Arabidopsis*, functions as a repressor of flowering (Pin et al., 2010).

High temperature is one of the primary environmental stress factors that affect the normal growth and development of plants (Chen et al., 2017). Studies have found that environmental temperature regulation of plant flowering is affected by multiple pathways. At ambient temperatures, *FLC* expression is quantitatively modulated by a chromatin-silencing mechanism, and the effect of chromatin on transcription and co-transcriptional processing is of central importance to the regulation of gene expression. The FT protein, acting as a florigen, is translocated from mature leaves to the shoot apex, simultaneously forming a complex with FLOWERING LOCUS D (FD). *SOC1* is first evoked in the shoot apex during the floral switch process (Kong et al., 2016). The FT-FD dimer and *SOC1* activate the downstream genes *LEAFY* (*LFY*), *AP1*, and *FRUITIFUL* (*FUL*) to define floral characteristics. *FLC* interacts with *SHORT VEGETATIVE PHASE* (*SVP*) to inhibit *SOC1* transcription by directly combining with the *SOC1* promoter in the vegetative phase, among which *SVP*, *FLM*, and their homologous genes play a key role (Balanzà et al., 2014). *SVP* is a flowering suppressor that inhibits plant flowering by combining with *FT* and *SCO1* gene promoter elements. The autonomous regulatory factors *FCA* and *FVE* participate in the environmental temperature regulation pathway by inhibiting *SVP* (Kong et al., 2016). In *Arabidopsis*, high temperatures reduce SVP-FLC and promote flowering (Jones et al., 2020). *SOC1* contributes to the GA-regulated floral transition, and *SOC1* expression is increased in response to GA treatments. Studies have shown that the *LsFT* gene is involved in the process of high temperatures promoting bolting and flowering in leaf lettuce, and using RNA interference (RNAi) technology to knock down *LsFT*, the *LsSOC1* gene leads to delayed bolting and insensitivity to high temperatures (Chen et al., 2017, 2018).

Melatonin (*N*-acetyl-5-methoxytryptamine) was first discovered in plants in 1995 (Kanwar et al., 2018), and whether melatonin is universally present and what its function is in plants has subsequently attracted widespread attention. The presence of melatonin was reported in all plants tested and in different parts of plants, including roots, stems, leaves, flowers, fruits, and seeds (Arnao and Hernández-Ruiz, 2020; Onik et al., 2020), where the focus was on the effects of exogenous melatonin on plant growth, morphogenesis, and certain environmental stresses (Sun et al., 2020). Melatonin’s actions include its ability to act as a plant biostimulator against stress, both biotic and abiotic, its ability to regulate plant growth, and its ability to regulate the processes of plant vegetative development (Arnao and Hernández-Ruiz, 2018, 2019; Jahan et al., 2021a). In studies of senescence in kiwifruit leaves, due to the enhanced scavenging activity of the antioxidant enzymes peroxidase (POD), superoxide dismutase (SOD), and catalase (CAT), membrane damage was reduced, and reduction in the hydrogen peroxide (H_2_O_2_) content effectively delayed senescence (Liang et al., 2018). In general, cold, heat, salinity, drought, UV radiation, and chemical toxicity are countered or mitigated by the presence of melatonin (Arnao and Hernández-Ruiz, 2018; Choi and Back, 2019; Moustafa-Farag et al., 2019). Melatonin promotes the relationship between the growth of maize seedlings and the pathways related to nitrogen metabolism, coordinating the growth and development of maize seedlings and resulting in increased plant survival rates, higher shoot and deeper root growth, and improved photosynthetic efficiency (Erdal, 2019). Melatonin also promotes the relationship between grape seedling growth and sugar metabolism, improving the resistance of grape seedlings, accompanied by improved chloroplast and stomatal morphologies (Zhong et al., 2020).

In this study, in order to obtain insights into transcriptional regulation by melatonin treatment on early bolting and flowering in leaf lettuce under high-temperature stress, we used RNA sequencing (RNA-seq) analysis to study exogenous melatonin at high temperatures in the bolting-sensitive lettuce cultivar “S39.” Transcriptome analysis revealed that plant hormone signals, including ABA, ethylene, and GA signals, are involved in this process. The level of *LsMYB15*, an ABA-related gene, increases significantly during this process. Based on an analysis of the genes in the stem length-related gene module and using weighted gene co-expression network analysis (WGCNA), we determined that *LsMYB15* plays an important regulatory function in bolting in leaf lettuce. We propose that *LsMYB15* could be a useful and effective genetic resource for the improvement of leaf lettuce quality.

## Materials and Methods

### Plant Materials and Growing Conditions

The bolting-sensitive *L. sativa* cultivar S39 was selected and grown at the Beijing University of Agriculture Experimental Station under greenhouse conditions [seeds provided by Cathay Green Seeds (Beijing) Co., Ltd.]. The photoperiod was 16 h/8 h (light/dark), the light intensity was maintained at 200 μmol m^−2^ s^−1^, the temperature was maintained at 20°C/15°C (day/night), and the relative humidity was 60–65%. Pest control and water management were carried out according to standard practices. A total of 300 uniform and disease-free plump seeds were selected, which were then immersed in 9 cm-diameter petri dishes filled with filter paper moistened with distilled water. The dishes were then placed in a lighted incubator in order to germinate. Following the development of a white radical, the seeds were transplanted into a plug tray and placed in a lighted incubator for cultivation.

### Melatonin Treatment

When the leaf lettuce seedlings had reached the three-leaf-center stage, they were transplanted to a nutrition bowl 11 cm (diameter) × 12 cm (height), which remained in the light incubator; plants displaying consistent growth conditions were selected for testing. In October 2020, when the seedlings reached the five-leaf-center stage, they were subjected to a day/night temperature setting of 20°C/15°C, a photoperiod of 16 h/8 h, a light intensity of 200 μmol m^−2^ s^−1^, and a relative humidity of 60–65%. Following initiation of the treatment, melatonin (Sigma-Aldrich, Shanghai, China) was sprayed onto the plants with a small sprayer at 9:00 a.m. on the morning of treatment at concentrations of 0, 10, 50, 100, and 150 μmol L^−1^. Wearing gloves, the spray was applied evenly to leaf surfaces until the surfaces were wet but not dripping. Exogenous melatonin was applied every 3 days, and following treatment for 15 days, the plants were sampled at approximately 9:00 a.m., we selected leaves that had been subjected to different concentrations of melatonin in order to determine the physiological indicators of the indices of the primary functional leaves.

In February 2021, when the seedlings had reached the five-leaf-center stage, they were subjected to a day/night temperature setting of 35°C/30°C, a photoperiod of 16 h/8 h, a light intensity of 200 μmol m^−2^ s^−1^, and a relative humidity of 60–65% for high-temperature treatment. Following initiation of the heat treatment, solutions of 0 μmol L^−1^ melatonin (H) and 100 μmol L^−1^ melatonin (HM) were sprayed onto the plants using the same small sprayer at 9:00 a.m. Again wearing gloves, the spray was applied evenly to the leaf surfaces until the surfaces were wet but not dripping. Exogenous melatonin was applied every 3 days, and the plants were subjected to high-temperature treatment for 30 days and sampled at approximately 9:00 a.m. The leaf lettuce was then immediately frozen in liquid nitrogen and stored at 80°C for further analysis. We selected leaves (in triplicate) at 0, 6, 9, 15, 18, and 27 days that showed obvious changes in phenotype, and the physiological indicators of the indices of the primary functional leaves were determined and subjected to RNA-seq analysis.

### RNA Extraction and qRT-PCR Analysis

An RNA extraction kit (Aidlab, Beijing, China) was used to extract total RNA from lettuce leaves according to the manufacturer’s instructions, and RNA was converted into complementary DNA (cDNA) using an Access RT-PCR system (Promega Corporation, Madison, WI, United States). qRT-PCR and SYBR Green qPCR Mix (Takara Bio Inc., Shiga, Japan) and a Bio-Rad CFX96 real-time PCR system (Bio-Rad, Hercules, CA, United States) were used to analyze gene expression levels. The US National Center for Biotechnology Information (NCBI) primer basic local alignment search tool (BLAST) algorithm^1^ was used to design PCR primers, which are listed in Supplementary Table 3. Next, 10 μl 2 × SYBR Green qPCR Mix (Takara Bio Inc.), 7 μl ddH_2_O, 1 μl upstream primer, 1 μl downstream primer, and 1 μl 100 ng cDNA were added to tubes, for a total volume of 20 μl. After mixing the reaction, the program was set to 95°C for 30 s, 95°C for 10 s, 60°C for 15 s, and 72°C for 30 s for 39 cycles. Following completion of the amplification cycle and cooling to 60°C, the DNA product was denatured by heating it to 95°C, and a melting curve was generated after the operation was completed. The leaf lettuce 18S ribosomal RNA gene was used as an internal control to normalize transcript levels, and 2^(−ΔΔ*C*t)^ analysis was used to calculate the transcript levels.

### RNA-Seq Library Preparation and Sequencing

RNA library preparation and sequencing were carried out. The preliminary quality inspection included Nanodrop detection, agarose gel electrophoresis detection, and Agilent 2100 detection, which were used to determine RNA concentration, RNA integrity, and RNA integrity number (RIN), respectively. A 1% agarose gel was used to test for RNA degradation and contamination. If the RNA bands were clear, it was taken that there were no impurities or contamination. The RNA 6000 Nano kit was used for testing, the kurtosis of the sample was concentrated, and the RNA degradation rate was low, generally meeting the requirements for standard library construction. According to the recommendation of the manufacturer (Shanghai Majorbio Biopharm Technology Co., Ltd), 33 sequencing libraries were generated, the libraries were sequenced on an Illumina HiSeq platform (Illumina, Inc., San Diego, CA, United States), and paired-end reads were generated. SeqPrep software^2^ and Sickle^3^ were used to perform quality control on the original sequencing data to obtain high-quality quality control data (clean data) to ensure the accuracy of subsequent analytic results.

### Differential Expression Analysis

After obtaining the read counts of genes, the differential expression of genes between samples was analyzed to identify differentially expressed genes (DEGs) between samples, and the functions of differential genes were investigated. The differential expression module selects the DESeq2 software to analyze the raw counts. The default parameters are *p* < 0.05 & |log2FC| > = 1; to control the probability or frequency of errors in the overall inference result, the *p* value obtained by the statistical test was subjected to multiple test corrections, which is known as the *p*-adjust value. The platform uses BH (FDR for correction with Benjamini/Hochberg) for multiple test corrections of data. At values of *p* < 0.05, genes were considered differentially expressed.

### GO and KEGG Cluster Analysis of Differentially Expressed Genes

The gene ontology (GO) database^4^ was used to analyze genes and gene products according to their participation in biological processes (BPs), molecular functions (MFs), and cellular components (CCs), which were classified and annotated according to these three aspects. For *p* < 0.05, DEGs were considered significantly enriched. Kyoto Encyclopedia of Genes and Genomes (KEGG)^5^ to perform KEGG pathway enrichment analysis on the genes in the gene set. The calculation principle is the same as that used in the GO function enrichment analysis. Fisher’s exact test was used for enrichment analysis. The Benjamini–Hochberg (BH) multiple calibration method was chosen to check the *p* values. For corrected *p* < 0.05, the KEGG pathway that meets this condition was considered to be significantly enriched in the gene set.

### Co-expression Analysis of Transcription Factors and Phenotypic Data (WGCNA)

Weighted gene co-expression network analysis (WGCNA) was used to identify modules of highly correlated genes based on the FPKM data.^6^ After obtaining the gene modules that are commonly expressed, we linked the modules to the phenotypic information of interest to explore whether the expression of transcription factors is related to high-temperature bolting genes.

### Construction of and Infection With *LsMYB15* VIGS Vectors

The 340-bp open reading frames of *LsMYB15* were used to design the primers (Supplementary Table 3). The fragments were then cloned from the S39 cDNA. The cloned fragments and pTRV2 empty vector were digested with EcoR1 endonuclease and BmaH1 endonuclease. Following purification, the fragments were ligated and transformed to obtain a recombinant plasmid. The identified recombinant plasmid was transformed into the *Agrobacterium* strain GV3101, and an infection solution was prepared. The infection buffer consisted of 10 mM MgCl_2_, 10 mM MES, and 20 mM acetosyringone.

Three groups of plants were set up: a blank control group, in which the plants received no injection (WT); a negative control group, in which pTRV2 and pTRV1 empty vectors were mixed in a 1:1 ratio and injected into the plants (TRV2); and an experimental group, in which pTRV2-*LsMYB15* and pTRV1 empty vectors were mixed in a 1:1 ratio and injected (TRV2-*LsMYB15*; Wang et al., 2021). After 1 week of infection, the plants were held at 35°C for 1 week, and the other growth conditions remained unchanged. Plant morphological changes were monitored, including weekly measurements of stem length. After 1 week of high-temperature treatment, the new leaves that grew following the injection were randomly selected, then tested using the characteristic tobacco rattle virus (TRV) primers to determine whether the TRV virus had been transferred into the plants, and finally treated with exogenous melatonin. The cDNA of the young leaves was used to detect the effects by PCR and qRT-PCR using gene-specific primers. To observe morphological changes in the gene-silenced plants, the stem lengths of the plants that were infected for 1 week were measured.

### Enzyme-Linked Immunosorbent Assay

An Enzyme-Linked Immunosorbent Assay (ELISA) test kit (Thermo Fisher Technology Co., Ltd., Shanghai, China) was used for testing in accordance with the manufacturer’s instructions. The determination of antioxidant enzymes and the extraction and determination of endogenous hormones were both determined by ELISA. First, 0.1 g of lettuce leaves was weighed and ground into a powder using liquid nitrogen. The standard was then diluted, and samples were added according to the operating procedure and incubated at 37°C for 30 min. Each well was filled with a washing solution, incubated for 30 s, and the washing solution then discarded, which was repeated five times. A 50-μl volume of enzyme-labeled reagent was added to each well, except for blank wells, and incubated at 37°C for 30 min. The washing was repeated five times. Next, 50 μl of developer A and 50 μl of developer B were added to each well, the plate was gently shaken, and the color was developed at 37°C for 10 min in the dark. Finally, 50 μl of stop solution was added to each well to stop the reaction (in this case, the blue color turned yellow to indicate that the reaction had stopped). Measurements were then taken within 15 min. The blank well was adjusted to zero, and the absorbance/optical density (OD) of each well in sequence was measured at a wavelength of 450 nm. The OD values of plant ascorbate peroxidase (APX; EC 1.11.1.11), plant superoxide dismutase (SOD; EC 1.15.1.1), plant catalase (CAT; EC 1.11.1.6), plant peroxidase (POD; EC 1.11.1.7), phenylalanine ammonia lyase (PAL; EC 4.3.1.5), and plant polyphenol oxidase (PPO; EC 1.10.3.1) were measured at 290, 560, 240, 450, 290, and 420 nm, respectively. The ODs increased by 0.01, 0.01, 0.01, 0.01, 0.01, and 0.001 units (U), respectively.

### Data Analysis

Experimental data were analyzed using one-way ANOVA followed by Tukey’s multiple range test to compare differences between the significance of the difference was *p* ≤ 0.05 or *p* ≤ 0.01. Origin 95, Microsoft Excel 2016 and IBM SPSS Statistics 22 were used for analysis.

## Results

### Effects of Treatment With Different Concentrations of Melatonin on Growth and Development in Leaf Lettuce

**Figure 1:**
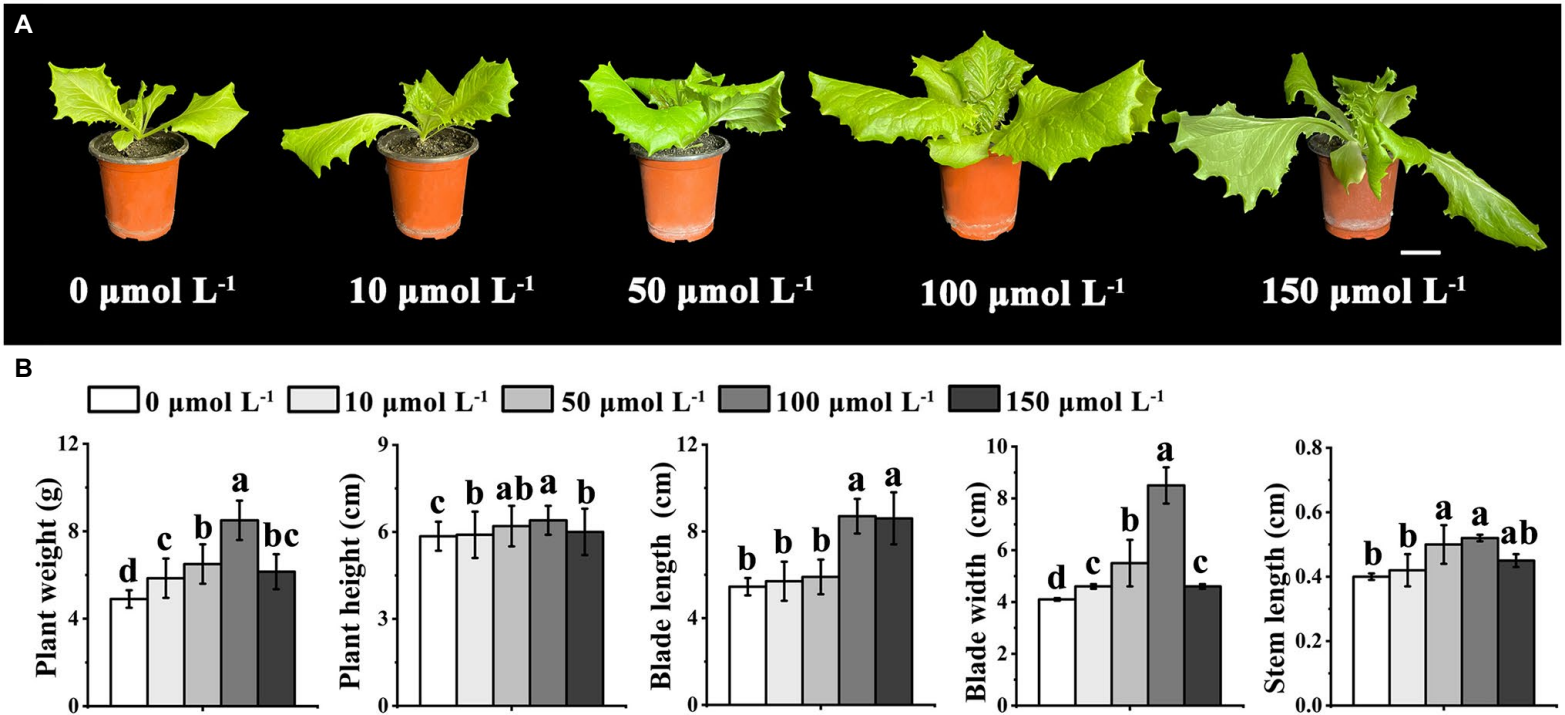
Effects of different concentrations of melatonin treatment under control temperature (20°C) on growth and development in leaf lettuce. **(A)** Leaf lettuce of the bolting-sensitive line S39 treated with 0, 10, 50, 100, and 150 μmol L^−1^ melatonin, corresponding to the morphological features of five different concentrations. Scale bars = 5 cm. **(B)** The weight, height, blade length, blade width, and steam length of leaf lettuce in five different concentrations of melatonin. Different letters above the bars indicate significantly different values (*p* < 0.05) calculated using one-way analysis of variance (ANOVA) followed by a Tukey’s multiple range test.

To examine the potential role of melatonin in regulating the bolting-sensitive plant line S39, we treated plants with different concentrations of exogenous melatonin (0, 10, 50, 100, and 150 μmol L^−1^), and then housed them under controlled-temperature conditions (20°C) for 15 days (Figure 1A). The results showed that exogenous melatonin significantly altered the blade length and blade width of leaf lettuce (Figure 1B). Specifically, a melatonin concentration of 100 μmol L^−1^ increased the leaf length and leaf width of leaf lettuce by 45 and 52%, respectively, compared to those treated with the lower concentrations. Therefore, we inferred that exogenous melatonin affected the growth and development of leaf lettuce. The preliminary experiment indicated that 100 μmol L^−1^ melatonin significantly enhanced growth vigor compared to the other treatment groups, and the plants grew very well. However, a melatonin concentration of 150 μmol L^−1^ resulted in serious wilting. To verify whether melatonin also affected the growth and development of leaf lettuce under high-temperature conditions, we selected 100 μmol L^−1^ melatonin for subsequent studies.

### Transcriptome Analysis of Leaf Lettuce Treated With Melatonin and Subjected to High Temperatures

**Figure 2:**
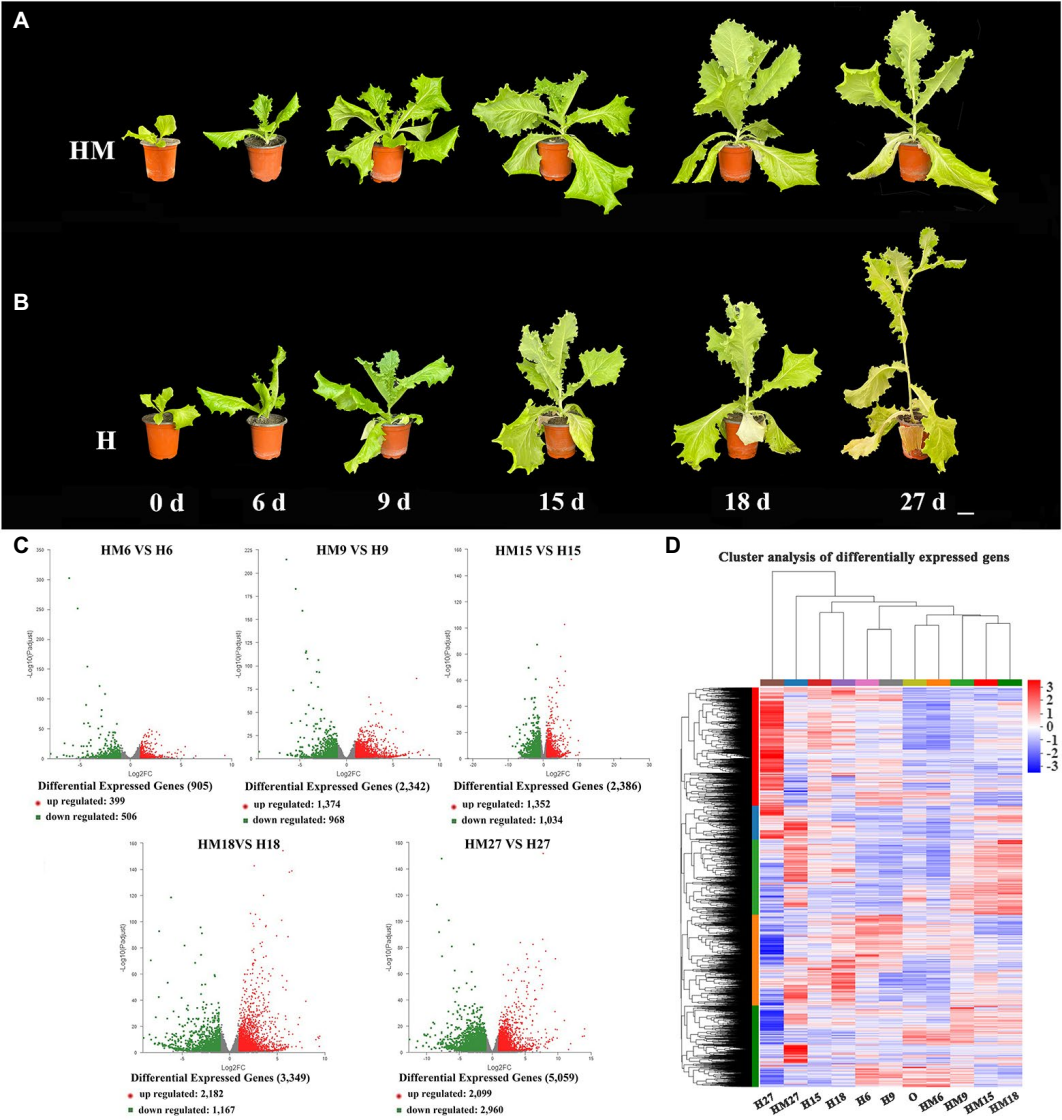
RNA-seq analysis of leaf lettuce treated with 100 μmol L^−1^ melatonin at high temperature during the growth period (the control group did not receive exogenous melatonin under the same conditions). **(A)** In 100 μmol L^−1^ melatonin treatment (HM), the leaf developmental stages of phenotypes changed significantly at 0, 6, 9, 15, 18, and 27 days with different stem lengths, and whole plants were characterized. Scale bars = 5 cm. **(B)** In the control group that did not have exogenous melatonin (H), the leaf developmental stages of phenotypes changed significantly on 0, 6, 9, 15, 18, and 27 days with different stem lengths, and whole plants were characterized. Scale bars = 5 cm. **(C)** Volcano plot visualizing differentially expressed genes (DEGs). The DEGs are shown in red and green. The *x*-axis represents the fold change in HM6 vs. H6, HM96 vs. H9, HM15 vs. H15, HM18 vs. H18 and HM27 vs. H27 (on a log2 scale), and the *y*-axis represents the negative log10-transformed *p* values (*p* < 0.05) of the t test for determining differences between the samples. **(D)** Cluster analysis of DEGs during different leaf treatments.

Leaf lettuce is susceptible to high-temperature stress during cultivation, which causes bolting and flowering, reducing the yield. Therefore, we treated plants with 100 μmol L^−1^ melatonin to further observe whether melatonin affected the growth of lettuce under high-temperature conditions. Furthermore, we treated leaf lettuce with 100 μmol L^−1^ melatonin at high temperatures during the growth period (the control group was not treated with exogenous melatonin under the same conditions) and found that phenotypes changed significantly at 0, 6, 9, 15, 18, and 27 days. Compared to the new phenotype, under the high-temperature treatment, the control group without exogenous melatonin bolted significantly faster than the treatment group with exogenous melatonin. Moreover, exogenous melatonin-treated lettuce leaves were larger and greener than those in the control group (Figures 2A,B). Therefore, we selected the S39 cultivar for transcriptome analysis in order to understand the exogenous melatonin regulation network of bolting during leaf lettuce development at high temperatures. Total RNA was extracted from three biological replicates of six different leaf developmental stages of S39 and used to generate cDNA libraries, which were subjected to paired-end sequencing using Illumina high-throughput RNA sequencing. After removing reads derived from rRNA and those of low quality, the total length of clean reads ranged from 45,664,626 to 63,758,324 among the different libraries, and almost 67% of the sequenced reads could be aligned to the apple genome (Supplementary Table 1). A Pearson correlation analysis indicated that the three libraries from the biological replicates of each developmental stage had highly consistent transcriptome profiles (Supplementary Figure 1).

### Identification of Genes With Differential Expression During Leaf Development Under Melatonin Treatment

Fragments per kilobase of transcript per million mapped reads (FPKM) values were used to investigate transcript differences. As a result, 399 genes (ratio > 2.0, *p* < 0.05) were found to be upregulated and 506 genes (ratio < 0.5, *p* < 0.05) were downregulated in HM6 vs. H6. The DEGs at this stage were the lowest; 1,374 genes (ratio > 2.0, *p* < 0.05) were upregulated and 968 genes (ratio < 0.5, *p* < 0.05) were downregulated in HM9 vs. H9; 1,352 genes (ratio > 2.0, *p* < 0.05) were upregulated and 1,034 genes (ratio < 0.5, *p* < 0.05) were downregulated in HM15 vs. H15 (Figures 2C,D); 2,182 genes (ratio > 2.0, *p* < 0.05) were upregulated and 1,167 genes (ratio < 0.5, *p* < 0.05) were downregulated in HM18 vs. H18. The expression level of upregulated differential genes was the highest, and 2,099 genes (ratio > 2.0, *p* < 0.05) were upregulated and 2,960 genes (ratio < 0.5, *p* < 0.05) were downregulated in HM27 vs. H27. The DEGs were the most abundant at this stage (Figures 2C,D). Therefore, we focused on the analysis of the HM27 vs. H27 stage with the highest expression of DEGs in order to determine the causes of leaf lettuce phenotypic changes.

**Figure 3:**
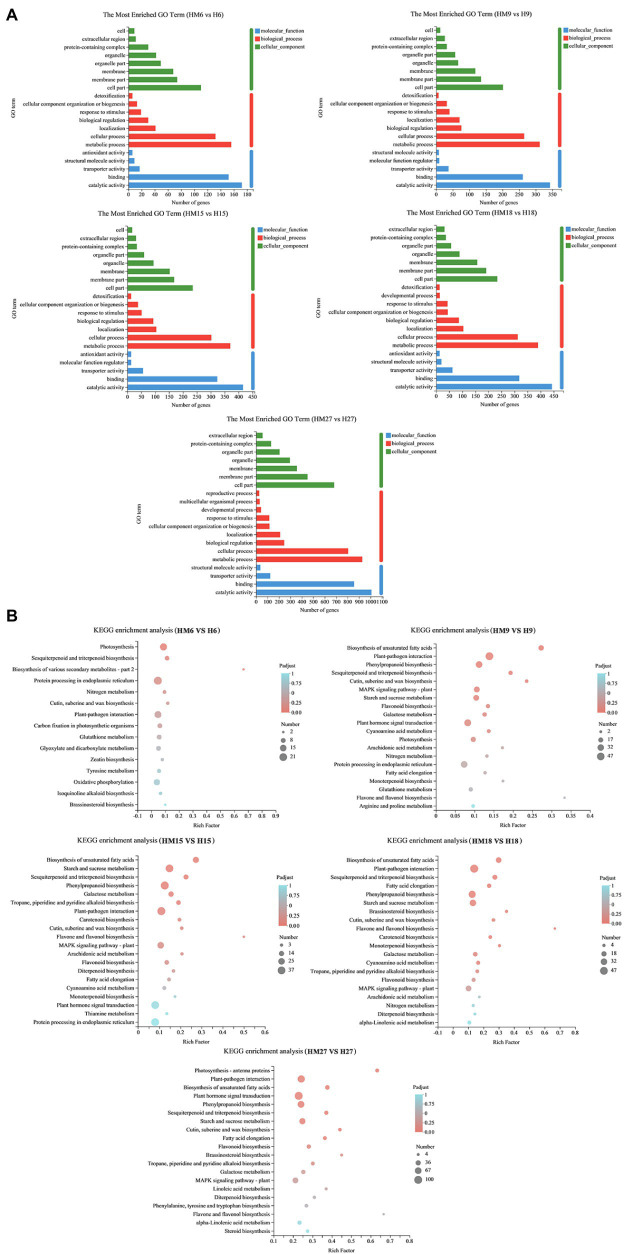
GO classification and KEGG pathway enrichment of differentially expressed genes (DEGs) in lettuce leaves. **(A)** GO classification of DEGs. **(B)** KEGG pathway enrichment of DEGs. “MH” represents 100 μmol L^−1^ melatonin treatment under high temperature, and “H” represents no exogenous melatonin treatment under high temperature.

DEGs (ratio > 2.0 or ratio < 0.5, *p* < 0.05) were classified into different functional categories based on their GO annotations. The numbers of DEGs involved in “metabolic processes” (MPs), BPs, “catalytic activity” (CA), and MF were largest in HM27 vs. H27 (Figure 3A). In all the treatments, most DEGs were from the BP and MF categories. KEGG is a pathway-related database that provides a classification for biologically complex patterns. Notably, KEGG terms associated with “plant hormone signal transduction,” “starch and sucrose metabolism,” and “phenylpropanoid biosynthesis” were enriched in HM27 vs. H27 and in HM9 vs. H9, which are stages that are significantly affected by variations in bolting (Figure 3B). Genes involved in “plant hormone signal transduction” were also enriched in HM27 vs. H27 and in HM9 vs. H9, including several plant hormone signal transduction and response proteins, including ABA-related genes, auxin-responsive genes, ethylene signal transduction pathway genes, and ethylene-responsive genes (Supplementary Table 2). These results suggested that, at high temperatures, exogenous melatonin not only plays an important role in leaf development but also affects bolting.

### Identification of Important Modules and Genes for Bolting Accumulation in Leaf Lettuce Using WGCNA Analysis

**Figure 4:**
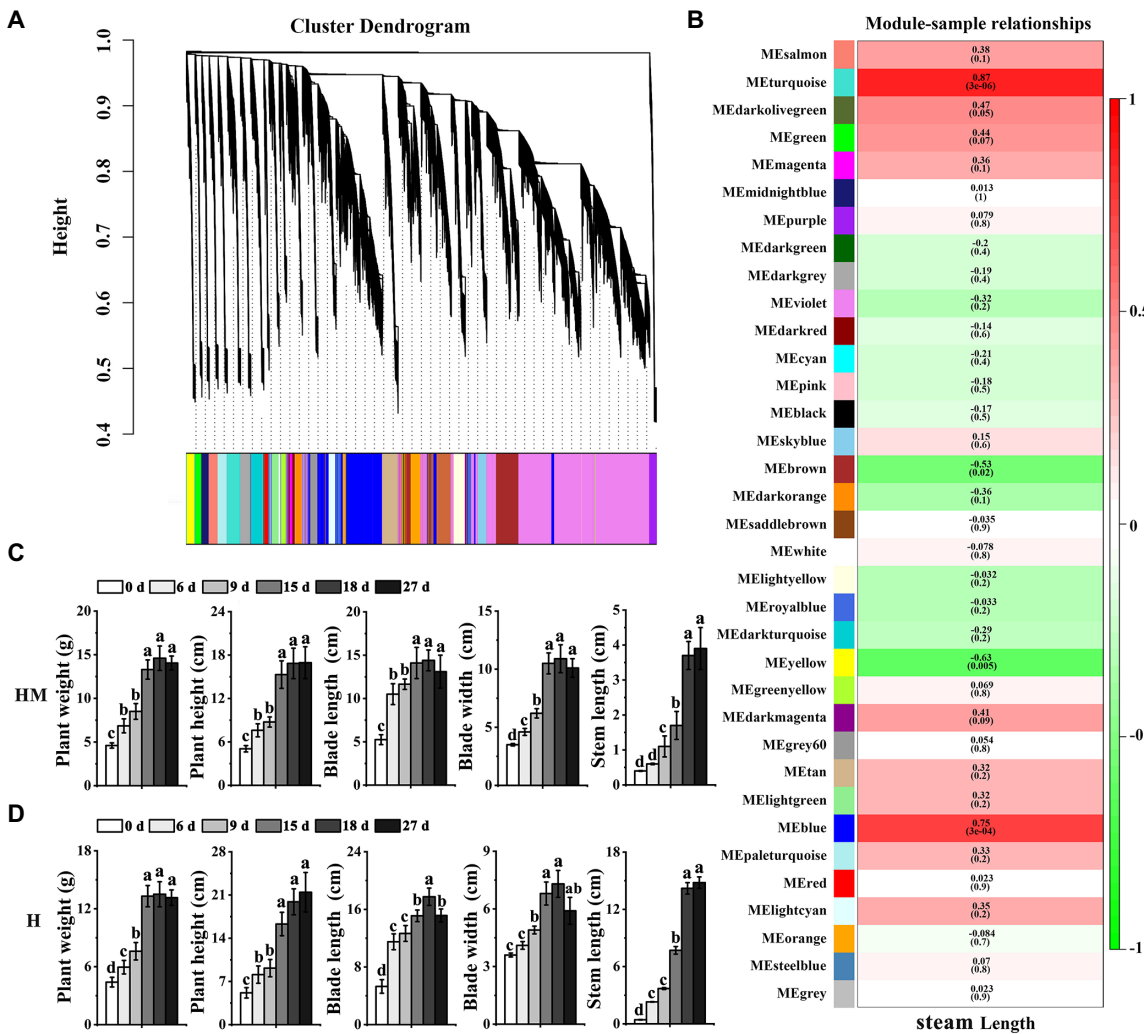
Network analysis dendrogram showing modules identified by weighted gene co-expression network analysis (WGCNA). **(A)** Dendrogram plot with color annotation. **(B)** Module-bolting correlations and corresponding *p* values (in parentheses). The left panel shows the seven modules. The color scale on the right shows module trait correlation from 1 (green) to 1 (red). **(C)** The weight, height, blade length, blade width, and steam length of leaf lettuce in five developmental stages with exogenous melatonin at high temperature. “MH” represents 100 μmol L^−1^ melatonin treatment under high temperature. **(D)** The weight, height, blade length, blade width, and steam length of leaf lettuce in five developmental stages without exogenous melatonin at high temperature. “H” represents no exogenous melatonin treatment under high temperature. Different letters above the bars indicate significantly different values (*p* < 0.05) calculated using one-way analysis of variance (ANOVA) followed by a Tukey’s multiple range test.

Weighted gene co-expression network analysis (WGCNA) is a method for identifying networks of genes with certain associated functions or traits and for revealing putative hub genes with particular influence (Horvath et al., 2008; Yang et al., 2018). To identify genes associated with bolting in leaf lettuce, we identified co-expressed gene sets by applying WGCNA (Figure 4A) to examine the genes expressed after excluding those with low FPKM values (average FPKM <1) and/or a low coefficient of variation (<1) across all development stages. The 38,915 DEGs in the five different treatment regimes that met these stringent criteria were categorized into 35 co-expression modules (Figure 4B).

Next, we measured the weight, height, blade length, blade width, and steam length of leaf lettuce. The steam length of leaf lettuce treated with melatonin at high temperatures was shorter than that not treated with melatonin (Figures 4C,D). An analysis of the relationship between modules and stem length revealed that the Turquoise (*r* = 0.87, *p* = 3e−06) module and the Blue module (*r* = 0.75, *p* = 3e−04) were highly correlated with bolting (Figure 4B).

Genes associated with the “protein processing in endoplasmic reticulum,” “ubiquitin-mediated proteolysis,” “photosynthesis-antenna proteins,” “MAPK signaling pathway-plant,” and the “phosphatidylinositol-signaling system” categories were enriched in the Turquoise and Blue modules. Additionally, genes in the “oxidative phosphorylation,” “glutathione metabolism,” “porphyrin and chlorophyll metabolism,” and “carotenoid biosynthesis” categories were also enriched in the Turquoise and Blue modules, which are all processes that have been associated with leaf lettuce bolting (Supplementary Figure 2).

**Figure 5:**
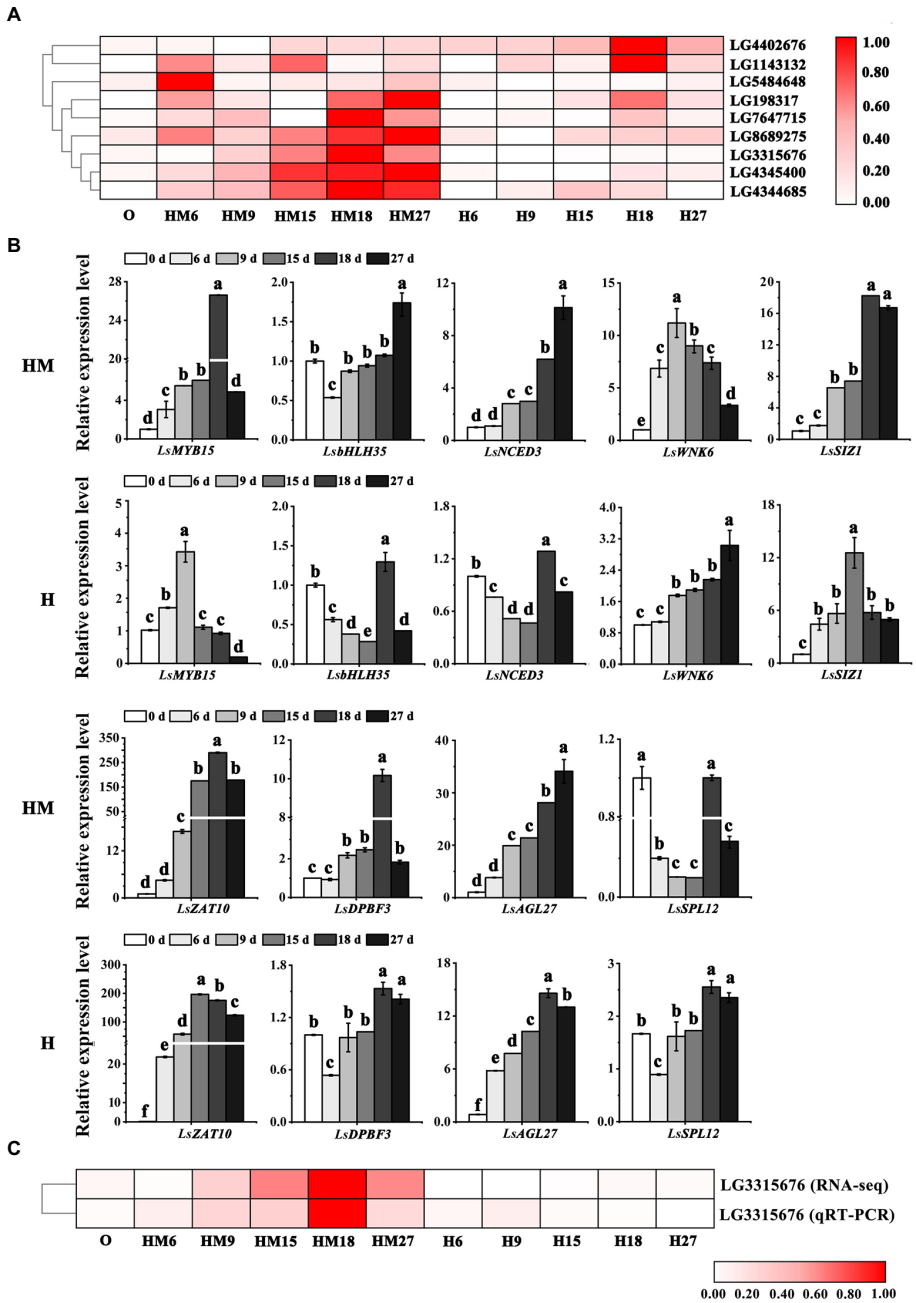
Identification and analysis of bolting-related genes. **(A)** Heatmaps describing the expression profiles of candidate genes related to bolting-related genes. **(B)** Validation of RNA-seq expression profiles *via* qRT-PCR. **(C)** Correlation analysis between ABA-related genes and expression of the related candidate *LsMYB15* (LG3315676) *via* RNA-seq and qRT-PCR data. “MH” represents 100 μmol L^−1^ melatonin treatment under high temperature, and “H” represents no exogenous melatonin treatment under high temperature. Different letters above the bars indicate significantly different values (*p* < 0.05) calculated using one-way analysis of variance (ANOVA) followed by Tukey’s multiple range test.

To validate expression of the DEGs and bolting-related genes from the Turquoise and Blue modules, qRT-PCR in leaf lettuce was carried out for nine representative genes. The expression levels of the bolting-related genes *LsMYB15* (LG3315676, XP_023766862.1), *LsbHLH35* (LG5484648, PLY83907.1), *LsZAT10* (LG198317, XP_023764915.1), *LsNCED3* (LG7647715, PLY98266.1), *LsDPBF3* (LG8689275, XP_023768520.1), *LsWNK6* (LG4344685, XP_023729088.1), *LsSIZ1* (LG1143132, XP_023733270.1), *LsSPL12* (LG4402676, XP_023741755.1), and *LsAGL27* (LG4402879, XP_023741806.1) were examined. A correlation analysis showed that the expression of these nine genes was closely related to bolting (Figure 5A). The DEGs between the control and melatonin groups showed that, following melatonin treatment, significant upregulation of the expression of most regulatory genes in the ABA-related genes occurred, except for the *LsSPL12* gene (Figure 5B), as determined by RNA-seq. The relative gene expression analysis of selected DEGs (*LsMYB15*, LG3315676) indicated high integrity and correlation between the transcriptome analyses from the RNA-seq and qRT-PCR results (Figure 5C). This result indicated that inhibiting bolting in leaf lettuce was positively affected by melatonin treatment as a result of it increasing the expression of ABA-related genes.

### Silencing of *LsMYB15* Expression in Leaf Lettuce Alters the Occurrence of Bolting

Under drought or cold conditions, ABA is often recruited as the primary signal for increasing the transcription levels of stress-responsive genes that may confer protection to assaulted plants (Agurla et al., 2018; Li et al., 2021). MYB transcription factors have been found to play important roles in many physiological processes under normal and adverse growth conditions (Guo et al., 2017). MYB15 is a member of the R2R3 MYB family of transcription factors in *Arabidopsis* (Ding et al., 2009). However, the role of MYB15 in high-temperature stress has not been previously reported. In the work reported below, we provide evidence showing that silencing of *LsMYB15* leads to bolting in leaf lettuce. To further elucidate the role of the *LsMYB15* genes, we suppressed their expression in leaves of the bolting-sensitive S39 lettuce cultivar using virus-induced gene silencing (VIGS) in the TRV vector.

**Figure 6:**
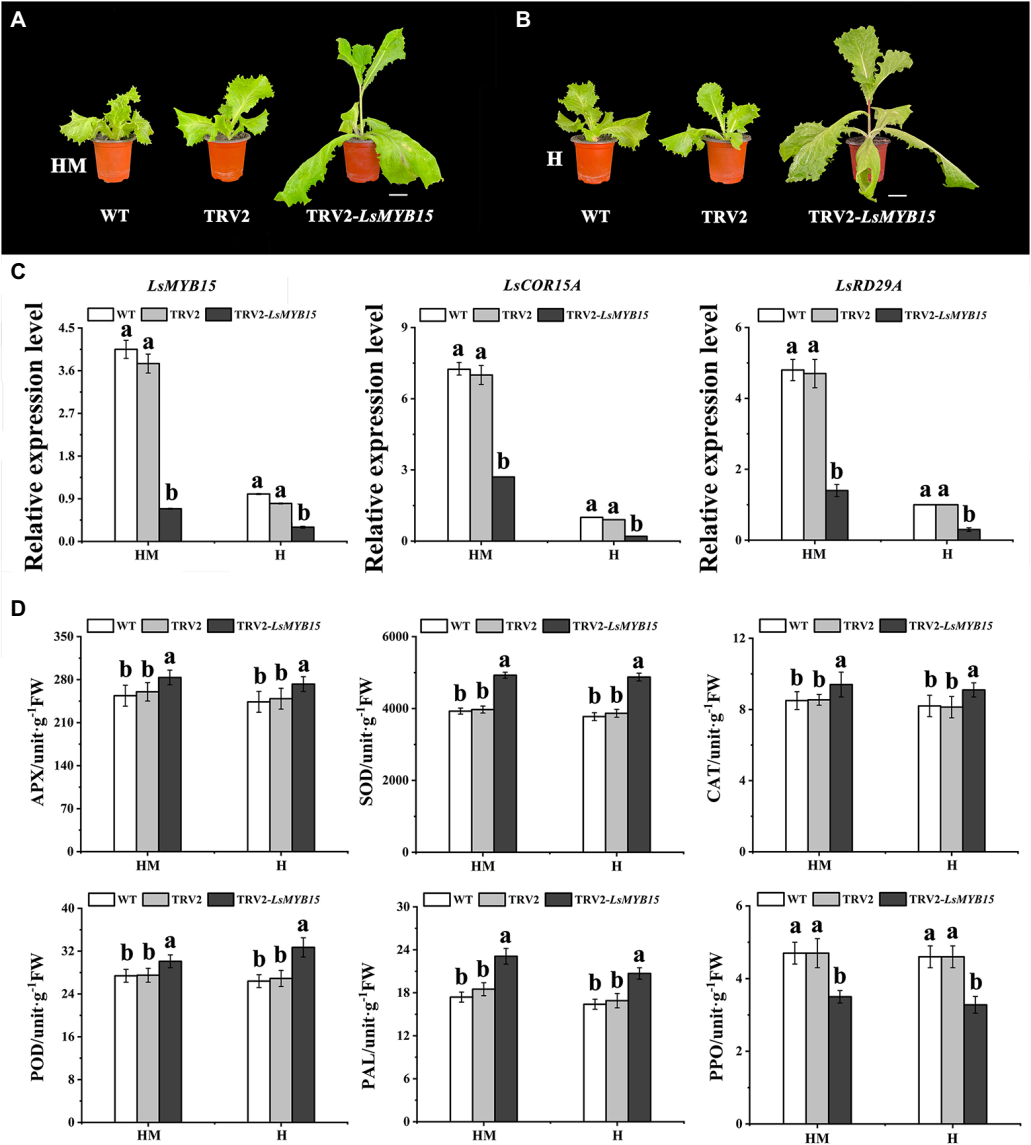
Silencing of *LsMYB15* genes in the bolting-sensitive line S39. **(A)** Under high-temperature conditions, the exogenous melatonin administration constructs developed a bolting phenotype 7 days postinfection. Scale bars = 5 cm. **(B)** Under high-temperature conditions, the control group that did not have exogenous melatonin construct developed a bolting phenotype 7 days postinfection. Scale bars = 5 cm. **(C)** Relative expression levels in inoculated TRV2-*LsMYB15* plants were determined using qRT-PCR to determine transcription levels of three genes (*LsMYB15*, *LsCOR15A*, and *LsRD29A*) in leaf lettuce plants. **(D)** Activities of APX, SOD, CAT, POD, PAL, and PPO during heat treatment of control and melatonin-treated leaf lettuce. Bars indicate the standard error from each mean of three independent replications. Mean values with the same letter are not significantly different (*p* < 0.05). An increase in absorbance of 0.01 at 290 nm, 0.01 at 560 nm, 0.01 at 240 nm, 0.01 at 450 nm, 0.01 at 290 nm and 0.001 at 420 nm per min was defined as one unit (U) of APX, SOD, CAT, POD, PAL, and PPO activity, respectively. “MH” represents 100 μmol L^−1^ melatonin treatment under high temperature, and “H” represents no exogenous melatonin treatment under high temperature. Error bars indicate the SEM of three replicate measurements. Different letters above the bars indicate significantly different values (*p* < 0.05) calculated using one-way analysis of variance (ANOVA) followed by Tukey’s multiple range test.

The leaf lettuce plants infiltrated with the virus containing TRV2-*LsMYB15* under high-temperature conditions and exposed to exogenous melatonin administration, along with a control group that did not receive exogenous melatonin or the viral construct, developed a bolting phenotype 7 days post-infection. In contrast, plants infiltrated with the empty vector pTRV2 did not bolt (Supplementary Table 3). The rapid bolting in plants expressing TRV2-*LsMYB15* was then examined in the control (Figures 6A,B). qRT-PCR revealed that the TRV2-*LsMYB15* transcript levels in the *LsMYB15*-silenced plants decreased by approximately 90% compared to the control plantlets. From the above phenotypic and physiological analyses, the expression levels of ABA-related genes in *LsMYB15*-silenced plants are of great significance. For this purpose, qRT-PCR was used to analyze and compare the gene transcription levels between the *LsMYB15*-silenced plants and the control plants (Supplementary Table 4). Based on the results from three independent analyses, we found that the transcription levels of three genes (*LsMYB15*, *LsCOR15A*, and *LsRD29A*) in the *LsMYB15*-silenced plants were significantly reduced in the presence of exogenous melatonin compared to the melatonin-treated control plants treated similarly. The transcript levels of *LsMYB15*, *LsCOR15A*, and *LsRD29A* were also significantly lower in the *LsMYB15*-silenced plants than in the control group in the absence of exogenous melatonin (Figure 6C). Taken together, we further hypothesized that *LsMYB15* plays a role in transcriptional regulation *via* modulation of bolting and indirect gene regulation, which could be the cause of the altered bolting in leaf lettuce.

The results of antioxidant enzyme activity measurements showed that APX, SOD, CAT, POD, and PAL activities exhibited a steady increase in both the control plants and the melatonin-treated leaf lettuce, with no significant difference between WT and TRV2, but PPO activity decreased rapidly (Figure 6D).

## Discussion

### Effects of Melatonin on Leaf Lettuce Under High-Temperature Stress

Melatonin is considered a potential plant growth regulator that enhances plant growth. Nevertheless, the role of melatonin in mediating the stress response in different plant species and growth cycles still needs to be explored. Melatonin can enhance the tolerance of plants to abiotic stresses such as drought, salt, ambient temperature, and heavy metals in different ways (Wang et al., 2017a,b; Sun et al., 2020). For example, in rice seedlings, exogenous melatonin treatment under salt stress increased the net photosynthetic rate of rice and enhanced the absorption and transmission of light energy, increasing the relative water content and sucrose and starch contents and improving the salt tolerance of rice (Yan et al., 2021). In wheat (*Triticum aestivum* L.), the application of melatonin reduced oxidative stress by lowering thiobarbituric acid-reactive substances and H_2_O_2_ content, increasing the activity of antioxidant enzymes and of photosynthesis and carbohydrate metabolism and actively improving heat resistance (Iqbal et al., 2021).

In conclusion, melatonin is extensively involved in the plant stress response, regulating growth and development and protecting plants from abiotic stress. High temperature is one of the primary environmental stress factors that affect the normal growth and development of leaf lettuce. Leaf lettuce growth temperatures above 30°C result in early bolting, and bolting reduces both plant quality and yields (Chen et al., 2017). Therefore, we selected 100 μmol L^−1^ melatonin at high temperatures during the growth period (the control group was not treated with exogenous melatonin under the same conditions) for further research. We found that the growth of leaf lettuce recovered following melatonin treatment at high temperatures, while the speed of bolting was significantly faster in the control group than in the treatment group. In addition, exogenous melatonin-treated lettuce leaves were larger and greener than those in the control group, with the most significant leaf width being 49% higher than that of the control group (Figures 2A–D). These results demonstrated that melatonin is involved in the stress response of leaf lettuce, regulating its growth and development and protecting leaf lettuce from high-temperature stress.

### Plant Hormones Play an Important Role in Enhancing Plant Growth and Stress Resistance

Phytohormones participate in various processes throughout a plant’s lifecycle. Studies have shown that five classical plant hormones, including auxins, cytokinins, GAs, ABA, and ethylene, play important roles in plant growth and stress responses, as well as brassinosteroids, jasmonic acid, salicylic acid, strigolactones, and peptides (Wang et al., 2017a,b; Zhao et al., 2021). For example, in cucumber seedlings, indoleacetic acid—acting as a downstream signaling molecule—is involved in H_2_S-induced tolerance to chilling (Zhang et al., 2020). In *Arabidopsis*, high temperature promotes ABA accumulation, and elevated ABA levels trigger the upregulation of ABA enzyme biosynthesis genes, improving plant heat tolerance during growth (Gupta et al., 2020). In conclusion, hormones play a key role in adaptation to abiotic stress during plant growth and development. In this study, the number of DEGs involved in “plant hormone signal transduction” was significantly enhanced, as determined by KEGG analysis (Supplementary Figure 2). Using WGCNA, some of the genes screened from the high-correlation modules, including *MYB15*, *bHLH35*, *ZAT10*, *NCED3*, *DPBF3*, *WNK6*, *SIZ1*, and *SPL12*, were all ABA-related and have been reported to regulate plant growth and stress tolerance by regulating the ABA pathway (Figure 5). In perennial ryegrass (*Lolium perenne*) leaves, exogenous melatonin reduced the ABA content, delaying senescence. The reduction in ABA was correlated with downregulation of two ABA biosynthesis genes (*LpZEP* and *LpNCED1*), which were upregulated by heat stress, although melatonin suppressed this effect. Thus, the response of ABA in heat-induced senescence was delayed by melatonin through the reduction in ABA biosynthesis and the downregulation of signaling pathway factors (Zhang et al., 2017). We believe, therefore, that ABA plays an important role in the growth and development of lettuce under high-temperature conditions.

Melatonin exerts its effects by regulating various elements related to interfering with the activities of other phytohormones. For example, studies have found that, following exogenous melatonin treatment of tomato (*Solanum lycopersicum*) plants, the expression levels of ABA receptors induced by melatonin were increased and ABA signaling transduction pathways were activated, leading to heat resistance and inhibition of heat-induced senescence in tomato plants (Jahan et al., 2021b). In cucumber (*Cucumis sativus*) seedlings, exogenous melatonin were found to alleviate low-temperature stress by upregulating the expression of *CsZat12* and regulating the metabolism of PA and ABA (Zhao et al., 2017). In conclusion, ABA plays an important role in melatonin therapy and improves the tolerance of plants to abiotic stress. The results of this study demonstrate that melatonin regulates the growth and development of leaf lettuce by regulating the expression of ABA-related genes under high-temperature conditions, consistent with the results of previous studies.

### *LsMYB15* Regulates Bolting in Leaf Lettuce

The MYB transcription factor plays an important role in plant physiological processes under conditions of treatment with exogenous melatonin. In tea (*Camellia sinensis*) leaves, the increase in lignin content was found to parallel the rise in POD activity following melatonin treatment, which revealed that melatonin could increase lignin accumulation by enhancing POD activity. Additionally, melatonin also modified the expression of MYB transcription factor genes, which were regarded as candidates for the regulation of lignin metabolism in tea leaves (Han et al., 2021a,b). In water bamboo shoots, the transcription factors of *ZlMYB1* and *ZlMYB2* from the MYB family were increased, and melatonin treatment markedly reduced their expression, indicating that melatonin may be a participant in the transcriptional regulation of lignin synthesis (Yang et al., 2022). *LsMYB15* (LG3315676) was selected from the identified DEGs as a putative regulator of bolting using WGCNA. In *Arabidopsis*, *MYB15* is a positive regulator of drought and salt tolerance. Enhanced transcript levels of ABA biosynthesis, signaling, or responsive genes in *MYB15* overexpression lines also lead to increased tolerance to drought and salt stress (Ding et al., 2009; Li et al., 2021). When induced by low-temperature stresses, C-repeat binding factor (CBF) functions as a positive regulator to aid ABA-dependent increases in the expression levels of stress-responsive genes, leading to enhanced tolerance to freezing (Zhou et al., 2011). There were no changes in *COR15A* or *RD29A* in *MYB15*-overexpressing plants, suggesting that the coordination of upstream genes induces downstream genes more effectively with cellular protective functions (Chen et al., 2010). These studies suggest that melatonin treatment directly upregulates defense genes in response to stress through the ABA pathway, decreasing cellular injury.

In this study, *LsMYB15* silencing resulted in decreased expression levels of the downstream genes *LsCOR15A* and *LsRD29A*. These results indicate that *MYB15* may function differently and be involved in separate transcriptional regulation schemes under different stresses. From the results of the phenotypic, physiological, and molecular analyses conducted on leaf lettuce treated with exogenous melatonin under high-temperature conditions, strong positive correlations were observed among *LsMYB15*-silenced genes and reduced expression of the genes involved in ABA signaling (Figure 6). Furthermore, downstream stress–response pathways were activated, including those for reduced antioxidant systems, reduced osmoprotectants, enhanced membrane oxidation, and enhanced leaf senescence. In the presence of melatonin, leaf lettuce also bolted. Consequently, *LsMYB15* is likely to be a positive regulator of high-temperature bolting in leaf lettuce.

In conclusion, we have provided evidence that *LsMYB15* regulates bolting in leaf lettuce. These studies provide a more detailed understanding of the high-temperature stress regulatory network during leaf lettuce development and a new perspective for studying bolting and the potential applications of hormone treatment in agriculture.

## Data Availability Statement

The datasets presented in this study are publicly available, this data can be found here: https://www.ncbi.nlm.nih.gov/bioproject/PRJNA810911.

## Author Contributions

LC: data curation, formal analysis, methodology, and writing—original draft. MX: software, resources, and validation. CL: project administration, resources, software, and supervision. JH: methodology and validation. SF: supervision, resources, and funding acquisition. YH: conceptualization, writing—review and editing, and project administration. All authors contributed to the article and approved the submitted version.

## Funding

Financial support was provided by The National Natural Science Foundation of China (32172607) and the Beijing Municipal Organization Department-Top-notch Young Talents Program (2018000026833ZK76).

## Conflict of Interest

The authors declare that the research was conducted in the absence of any commercial or financial relationships that could be construed as a potential conflict of interest.

## Publisher’s Note

All claims expressed in this article are solely those of the authors and do not necessarily represent those of their affiliated organizations, or those of the publisher, the editors and the reviewers. Any product that may be evaluated in this article, or claim that may be made by its manufacturer, is not guaranteed or endorsed by the publisher.

## Footnotes

^1^ https://www.ncbi.nlm.nih.gov/tools/primer-blast

^2^ https://github.com/jstjohn/SeqPrep

^3^ https://github.com/najoshi/sickle

^4^ http://www.geneontology.org

^5^ http://www.genome.jp/kegg

^6^ https://horvath.genetics.ucla.edu/html/CoexpressionNetwork/Rpackages/WGCNA/
