# Supplementary material for: LsMYB15 Regulates Bolting in Leaf Lettuce (Lactuca sativa L.) Under High-Temperature Stress
Source: Front Plant Sci. 2022 Jun 28;13:921021. doi: 10.3389/fpls.2022.921021 (PMC9275828; doi:10.3389/fpls.2022.921021)
Supplement: Supplementary file 1 [file Data_Sheet_1.docx]

Supplementary Material

# Supplementary Figures and Tables

## Supplementary Figures


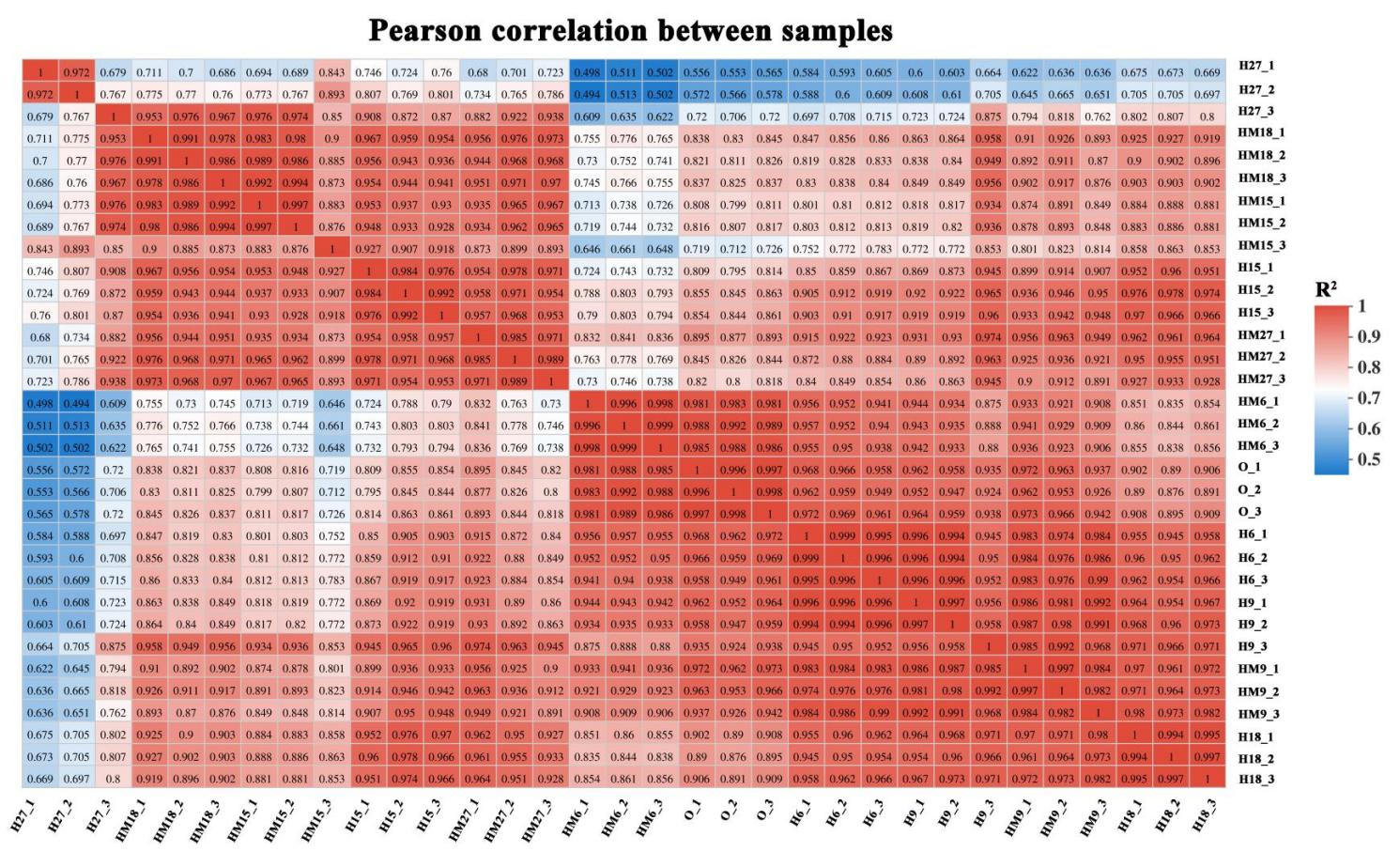


**Supplementary** **Figure 1.** Pearson correlation analysis. Heatmap of the correlations between biological replicates. PCC (Pearson correlation coefficient) values are quantitative indicators of the relative expression levels of all genes in each sample.

## Supplementary Figures


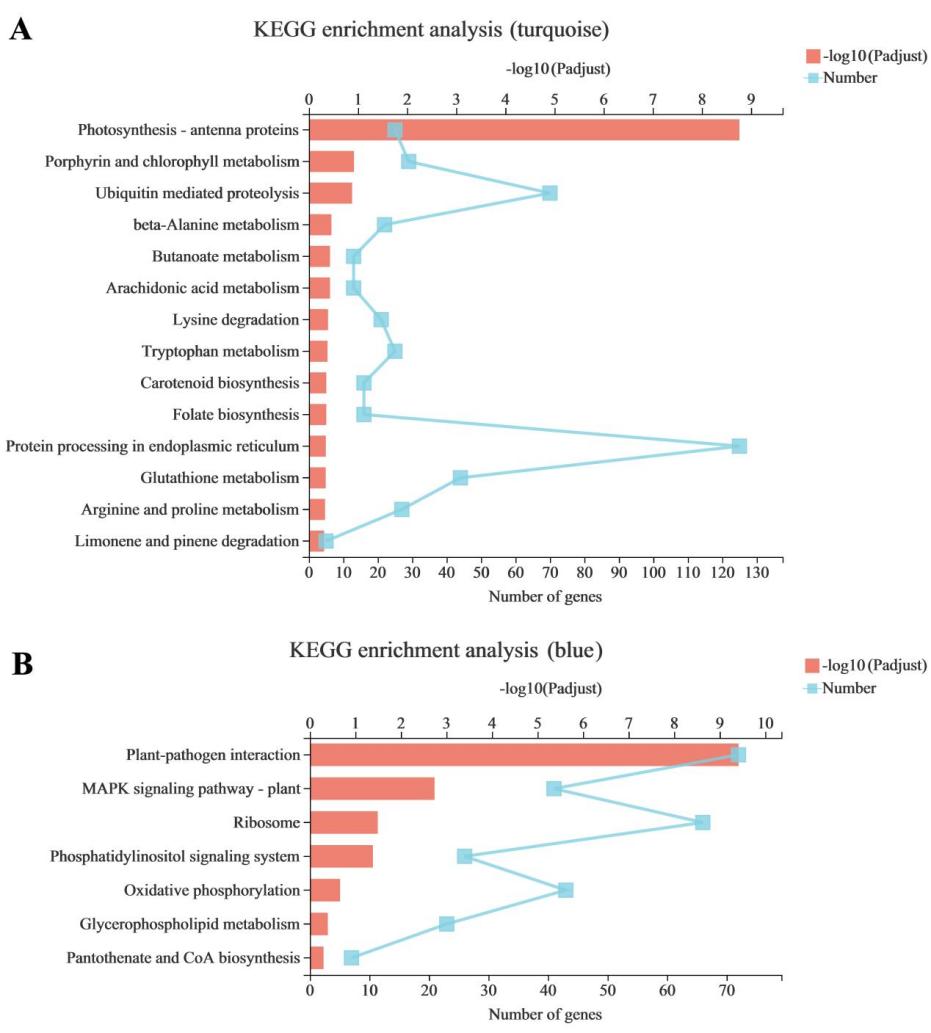


**Supplementary Figure 2.** KEGG (Kyoto Encyclopedia of Genes and Genomes) pathway enrichment of differentially expressed genes (DEGs) from the Turquoise and Blue modules. (A) KEGG pathway enrichment of the Turquoise module. (B) KEGG pathway enrichment of the Blue module. The x-axis represents the KEGG pathway, and the y-axis represents the number of genes/transcripts compared to this pathway, corresponding to different points on the broken line. The -log10-transformed P values (*P* < 0.05).

## Supplementary **Table**

**Supplementary Table 1**. Summary of RNA-Seq data from ‘S39’ leaf lettuce libraries.

| Sample | Raw reads | Clean reads | Clean bases | Error rate (%) | Q20 (%) | Q30 (%) | GC content (%) |
| --- | --- | --- | --- | --- | --- | --- | --- |
| O_1 | 53,210,062 | 52,835,290 | 7.80G | 0.02 | 98.35 | 94.8 | 45.87 |
| O_2 | 55,977,454 | 55,580,594 | 8.28G | 0.02 | 98.42 | 94.97 | 45.9 |
| O_3 | 47,879,268 | 47,558,696 | 7.04G | 0.02 | 98.4 | 94.91 | 45.7 |
| HT6_1 | 50,958,558 | 49,218,770 | 7.28G | 0.02 | 98.45 | 95.05 | 46.04 |
| HT6_2 | 54,034,122 | 45,664,626 | 6.76G | 0.02 | 98.36 | 94.83 | 46.17 |
| HT6_3 | 51,267,598 | 53,802,894 | 7.93G | 0.02 | 98.46 | 95.09 | 46.22 |
| HT9_1 | 50,219,676 | 53,887,248 | 7.96G | 0.02 | 98.39 | 94.9 | 45.31 |
| HT9_2 | 48,919,410 | 59,442,570 | 8.80G | 0.02 | 98.26 | 94.53 | 45.21 |
| HT9_3 | 48,575,998 | 49,076,618 | 7.23G | 0.02 | 98.47 | 95.11 | 45.17 |
| HT15_1 | 54,864,372 | 50,668,868 | 7.49G | 0.02 | 98.42 | 94.96 | 44.6 |
| HT15_2 | 54,380,080 | 54,818,086 | 8.12G | 0.02 | 98.42 | 94.97 | 44.63 |
| HT15_3 | 53,426,374 | 53,683,894 | 7.88G | 0.02 | 98.4 | 94.93 | 44.87 |
| HT18_1 | 49,421,850 | 48,626,450 | 7.19G | 0.02 | 98.35 | 94.74 | 44.48 |
| HT18_2 | 46,001,266 | 48,295,870 | 7.13G | 0.02 | 98.41 | 94.95 | 44.57 |
| HT18_3 | 54,158,510 | 57,830,066 | 8.49G | 0.02 | 98.44 | 95.03 | 44.71 |
| HT27_1 | 54,249,188 | 54,528,756 | 8.04G | 0.02 | 98.42 | 94.97 | 44.78 |
| HT27_2 | 49,408,744 | 53,102,568 | 7.86G | 0.02 | 98.39 | 94.86 | 44.56 |
| HT27_3 | 47,859,844 | 51,563,138 | 7.64G | 0.02 | 98.41 | 94.91 | 44.68 |
| H6_1 | 49,938,568 | 54,114,058 | 8.02G | 0.02 | 98.35 | 94.75 | 45.81 |
| H6_2 | 57,069,474 | 50,091,242 | 7.43G | 0.02 | 98.39 | 94.89 | 45.42 |
| H6_3 | 57,699,588 | 54,394,684 | 8.06G | 0.02 | 98.36 | 94.81 | 45.58 |
| H9_1 | 53,255,988 | 56,551,652 | 8.37G | 0.02 | 98.42 | 94.98 | 45.32 |
| H9_2 | 57,571,460 | 49,682,586 | 7.34G | 0.02 | 98.39 | 94.9 | 45.32 |
| H9_3 | 56,319,860 | 54,315,164 | 8.04G | 0.02 | 98.27 | 94.55 | 45 |
| H15_1 | 64,149,486 | 53,812,282 | 7.95G | 0.02 | 98.24 | 94.48 | 44.61 |
| H15_2 | 54,466,254 | 56,635,644 | 8.34G | 0.02 | 98.28 | 94.66 | 44.8 |
| H15_3 | 52,942,858 | 57,328,610 | 8.45G | 0.02 | 98.4 | 94.91 | 44.77 |
| H18_1 | 56,370,280 | 52,917,470 | 7.82G | 0.02 | 98.44 | 95.01 | 44.95 |
| H18_2 | 50,422,782 | 57,206,076 | 8.48G | 0.02 | 98.39 | 94.9 | 44.84 |
| H18_3 | 54,769,024 | 55,977,382 | 8.31G | 0.02 | 98.39 | 94.9 | 44.42 |
| H27_1 | 56,908,174 | 63,758,324 | 9.40G | 0.02 | 98.42 | 94.97 | 44.32 |
| H27_2 | 50,013,290 | 54,128,918 | 8.00G | 0.02 | 98.34 | 94.73 | 44.23 |
| H27_3 | 54,683,378 | 52,590,252 | 7.78G | 0.02 | 98.34 | 94.74 | 44.8 |

## Supplementary **Table**

**Supplementary Table 2.** Genes involved in ‘plant hormone signal transduction’.

| Gene ID | NR_hit-name | Name | Description |
| --- | --- | --- | --- |
| LG5505119 | XP_023748939.1 | ERS | ethylene receptor 2-like |
| LG5429360 | XP_023754086.1 | ERF1 | ethylene-responsive transcription factor 1B-like |
| LG3331750 | XP_023770527.1 | ERS | ethylene receptor 2-like |
| LG2210056 | XP_023767742.1 | ERF1 | ethylene-responsive transcription factor 1B-like |
| LG0106671 | XP_023755430.1 | ERF1 | ethylene-responsive transcription factor 1B-like |
| LG7605444 | XP_023737049.1 | SAUR | auxin-responsive protein SAUR71-like |
| LG1144905 | XP_023760881.1 | IAA | auxin-responsive protein IAA17-like |
| LG2183440 | XP_023764807.1 | SAUR | auxin-responsive protein SAUR50-like |
| LG2183440 | XP_023764807.1 | SAUR | auxin-responsive protein SAUR50-like |
| LG2247779 | XP_023739816.1 | SAUR | auxin-responsive protein SAUR21-like |
| LG2247811 | XP_023739808.1 | SAUR | auxin-responsive protein SAUR21-like |
| LG2247827 | XP_023739816.1 | SAUR | auxin-responsive protein SAUR21-like |
| LG2247831 | XP_023739779.1 | SAUR | auxin-responsive protein SAUR21-like |
| LG3281015 | XP_023749291.1 | SAUR | auxin-responsive protein SAUR50 |
| LG3289512 | XP_023744028.1 | SAUR | auxin-responsive protein SAUR32-like |
| LG3316496 | XP_023729948.1 | SAUR | auxin-responsive protein SAUR36-like |
| LG4352508 | XP_023770331.1 | IAA | auxin-induced protein IAA6-like |
| LG4352519 | XP_023770330.1 | IAA | auxin-responsive protein IAA15-like |
| LG4359812 | XP_023751346.1 | SAUR | auxin-responsive protein SAUR50-like |
| LG4359831 | XP_023751365.1 | SAUR | auxin-responsive protein SAUR21-like |
| LG5482970 | XP_023742510.1 | AUX1 | auxin transporter-like protein 2 |
| LG5491320 | XP_023737490.1 | IAA | auxin-responsive protein IAA14-like |
| LG5517878 | XP_023735591.1 | IAA | auxin-induced protein 22D |
| LG6590468 | XP_023750402.1 | ARF | auxin response factor 9-like isoform X1 |
| LG7622809 | XP_023757391.1 | ARF | auxin response factor 16-like |
| LG8687663 | XP_023755271.1 | SAUR | auxin-induced protein 15A-like |
| LG8687667 | XP_023755266.1 | SAUR | auxin-induced protein 15A-like |
| LG8687707 | XP_023755269.1 | SAUR | auxin-induced protein 15A-like |
| LG8687711 | XP_023755275.1 | SAUR | auxin-responsive protein SAUR19-like |
| LG8687727 | XP_023755264.1 | SAUR | auxin-responsive protein SAUR21-like |
| LG8687735 | XP_023755262.1 | SAUR | auxin-responsive protein SAUR21-like |
| LG8696106 | XP_023755283.1 | SAUR | auxin-induced protein 15A-like |
| LG8704030 | XP_023755261.1 | SAUR | auxin-responsive protein SAUR21-like isoform X2 |
| LG8707276 | XP_023736167.1 | AUX1 | auxin transporter-like protein 2 |
| LG8719869 | XP_023758635.1 | IAA | auxin-responsive protein IAA27 |
| LG8721640 | XP_023761393.1 | IAA | auxin-responsive protein IAA12-like |
| LG8729133 | XP_023765805.1 | SAUR | auxin-induced protein 6B |
| LG8738025 | XP_023730521.1 | IAA | auxin-responsive protein IAA20-like |
| LG8742572 | XP_023755280.1 | SAUR | auxin-responsive protein SAUR50-like |
| LG8761499 | XP_023750629.1 | AUX1 | auxin transporter-like protein 2 |
| LG9818968 | XP_023733419.1 | IAA | auxin-responsive protein IAA16-like |
| LG9818991 | XP_023733445.1 | IAA | auxin-induced protein 22D-like |
| LG2246404 | XP_023732062.1 | PYL | abscisic acid receptor PYL4-like |
| LG3302524 | XP_023736268.1 | PYL | abscisic acid receptor PYR1-like |
| LG7634878 | XP_023747489.1 | ABF | bZIP transcription factor 23-like |
| LG8701634 | XP_023760467.1 | PYL | abscisic acid receptor PYL4-like |
| LG8701954 | XP_023771032.1 | PYL | abscisic acid receptor PYL4-like |
| LG9796272 | XP_023749944.1 | PYL | abscisic acid receptor PYL4-like |
| LG9800597 | XP_023754714.1 | ABF | ABSCISIC ACID-INSENSITIVE 5-like protein 7 |
| LG9809662 | XP_023754710.1 | ABF | ABSCISIC ACID-INSENSITIVE 5-like protein 7 isoform X1 |

## Supplementary **Table 4.** After VGISS infection, the stem length of WT, TRV2, and TRV2-*LsMYB15* treated with and without melatonin significantly changed at high temperatures. Data were expressed as mean $\pm$ S.D. All statistical analyses were performed using SPSS (SPSS Inc., Chicago, IL, USA). Data were analyzed using independent t-tests at a significance level of *P* < 0.05 (*), or using one-way ANOVA Duncan’s multiple range tests at a significance level of *P* < 0.05.

| Name | WT | TRV2 | TRV2-*LsMYB15* |
| --- | --- | --- | --- |
| HM | 1.62$\pm$0.04 | 1.75$\pm$0.03 | 7.$13\pm$0.18** |
| H | 1.55$\pm$0.05 | 1.82$\pm$0.06 | 7.61$\pm$0.22** |

## Supplementary **Table**

**Supplementary Table 3****.** The primers used for qRT–PCR.

| Primers | Primer sequences | Used for |
| --- | --- | --- |
| q*LsMYB15*-F  q*LsMYB15*-R  q*LsbHLH35*-F  q*LsbHLH35*-R  q*LsZAT10*-F  q*LsZAT10*-R  q*LsNCED3*-F  q*LsNCED3*-R  q*LsDPBF3*-F  q*LsDPBF3*-R  q*LsWNK6*-F  q*LsWNK6*-R  q*LsSIZ1*-F  q*LsSIZ1*-R  q*LsSPL12*-F  q*LsSPL12*-R  q*LsAGL27*-F  q*LsAGL27*-R  q*LsCOR15A*-F  q*LsCOR15A*-R  q*LsRD29A*-F  q*LsRD29A*-R  q18SR-F  q18SR-R  TRV2-*LsMYB15*-F  TRV2-*LsMYB15*-R | 5′- ACACAACGCCGTGGAGTTTAGTC -3′  5′- ACTCAGTGCTGACCATGACAAATCC -3′  5′- CGTGGGAGAGAACACAGCATTGG -3′  5′- AGCAGTGATCTTGGCAGTGACAAC -3′  5′- GTTCTGGCTCTGGTTCCGATTCTG -3′  5′- TGCGTCTCCTGCGGTACAATTATAC -3′  5′- CCCAATTCCGAAGCCGAAGACG -3′  5′- TCGCCTCTAACTCCATTGTCATTGC -3′  5′- CGCCAGTCAAGCCTTACTCTTTCTC -3′  5′- CTCACCTAGTGTCGGTCGTCTCTC -3′  5′- AACCATACGAATCGCTGACCCAAG -3′  5′- CTGATAGTGCGGTGTCGGTGTTG -3′  5′- ATGGTGGTGGGCAGTTTGACTATTC -3′  5′- GCCACAGCCATAGCCTCATCTTC -3′  5′- GGAAATGGAAACGGAAACGGAAACG -3′  5′- CTAACTGATGGCGGAGGCGATTC -3′  5′- CGTGCTCTGCGATGTGGATGTC -3′  5′- GGTCCCGCTTTCTGGTTCAAGG -3′  5′- TCAGGAGCTGTTCTCACTGGTATGG -3′  5′- CGTCACCTTTAGCGGCGTAGATC -3′  5′- GGTGCGACTGATGAGGTGAAGC -3′  5′- GTGGAGCCAAGTGATTGTGGAGAC -3′  5'-GTGAGTGAAGAAGGGCAATG-3'  5'-CACTTTCAACCCGATTCACC-3'  5'-GATTCTGTGAGTAAGGTTACCGTTATCTTCGCCCAAACATCAAA-3'  5'-GAGACGCGTGAGCTCGGTACCGGGTCCAAAAGTCTCCACTCAAT-3' | qRT-PCR  qRT-PCR  qRT-PCR  qRT-PCR  qRT-PCR  qRT-PCR  qRT-PCR  qRT-PCR  qRT-PCR  qRT-PCR  qRT-PCR  qRT-PCR  qRT-PCR  qRT-PCR  qRT-PCR  qRT-PCR  qRT-PCR  qRT-PCR  qRT-PCR  qRT-PCR  qRT-PCR  qRT-PCR  qRT-PCR  qRT-PCR  Vector construction  Vector construction |
